# Emotional Prosodies Processing and Its Relationship With Neurodevelopment Outcome at 24 Months in Infants of Diabetic Mothers

**DOI:** 10.3389/fped.2022.861432

**Published:** 2022-05-18

**Authors:** Guoyu Sun, Yanan Liu, Rui Zhang, Cheng Peng, Yuehang Geng, Faliang Zhou, Xinlin Hou, Lili Liu

**Affiliations:** ^1^Department of Pediatrics, Peking University First Hospital, Beijing, China; ^2^Department of Pediatrics, Beijing Friendship Hospital, Capital Medical University, Beijing, China

**Keywords:** neonate, gestational diabetes mellitus, emotional prosody, neurodevelopment, event-related potential

## Abstract

Gestational diabetes mellitus (GDM) is one of the most common complications of pregnancy. Hyperglycemia of pregnancy is a risk not only for later obesity of the offspring but also do harm to their neurodevelopment from fetus. An ERP research has shown that children with autism spectrum disorder (ASD) was characterized by impaired semantic processing. In this study, we used event-related potential (ERP) to assess the procession of different emotional prosodies (happy, fearful, and angry) in neonates of diabetic mothers, compared to the healthy term infants. And to explore whether the ERP measure has potential value for the evaluation of neurodevelopmental outcome in later childhood. A total of 43 full-term neonates were recruited from the neonatology department of Peking University First Hospital from December 1, 2017 to April 30, 2019. They were assigned to infants of diabetic mothers (IDM) group (*n* = 23) or control group (*n* = 20) according to their mother's oral glucose tolerance test's (OGTT) result during pregnancy. Using an oddball paradigm, ERP data were recorded while subjects listened to deviation stimulus (20%, happy/fearful/angry prosodies) and standard stimulus (80%, neutral prosody) to evaluate the potential prognostic value of ERP indexes for neurodevelopment at 24 months of age. Results showed that 1) mismatch response (MMR) amplitudes in IDM group were lower than the control; 2) lower MMR amplitude to fearful prosody at frontal lobe was a high risk for increased Modified Checklist for Autism in Toddlers (M-CHAT) scores at 24 months. These findings suggests that hyperglycemia of pregnancy may influence the ability to process emotional prosodies in neonatal brain; it could be reflected by decreased MMR amplitude in response to fearful prosody. Moreover, the decreased MMR amplitude at the frontal lobe may indicated an increased risk of ASD.

## Introduction

Gestational diabetes mellitus (GDM) is one of the most common complications of pregnancy. The International Diabetes Federation (IDF) estimates that 16.8% of newborns were born from mothers with hyperglycemia during pregnancy, with 84% of maternal hyperglycemia being gestational diabetes mellitus (1). In China, with the change in childbearing attitudes and the implementation of the “bearing two-children” policy, the proportion of elderly pregnant mothers is increasing year by year, leading to a parallel increase in the prevalence of GDM. Infants of diabetic mothers (IDMs) are babies born to mothers with gestational diabetes and mothers who had pre-existing diabetes. In addition to the increased risk of obesity and metabolic syndrome in IDMs, current research also shows that hyperglycemia at pregnancy can interfere with fetal brain development. Mechanisms include a hyperglycemic environment during pregnancy, chronic intrauterine hypoxia due to hyperglycemia and hyperinsulinemia, iron deficiency, and docosahexaenoic acid (DHA) deficiency (2, 3). Consequently, children exposed to GDM in utero are at an increased risk for acquiring neurodevelopmental disorders including autism spectrum disorder (ASD) (4). ASD is a neurodevelopmental disorder with biological basis that manifests as persistent deficits in social communication and social interaction, as well as restricted and repetitive patterns of behavior, interests, and activities. Typical features appear in most children within 2 years of age (5–11). About $\frac{1}{4}$ to $\frac{1}{3}$ of children with ASD reach early language milestones, but regression or stagnation in language, communication, and/or social skills occurs at 15–24 months of age (12–17). Early diagnosis of these children is difficult due to the lack of specific neurological manifestations at birth and in the early postnatal period. In addition, due to the lack of specific medication, early identification and some early intervention may be helpful in improving the neurological prognosis of this group of children.

Emotional processing skills are extremely important in social interaction. However, children with ASD are unable to correctly understand the emotional responses of others (e.g., misinterpreting others' happiness as fear, failing to understand others' pain). They are also unable to share their interests or emotions (e.g., happiness, achievement, or apprehension) with social peers (e.g., caregivers or playmates) (18). Event-related potentials are cortical potentials generated during the brain's cognitive processing of stimulus information that can accurately reflect the brain's cognitive processing after stimulation. They also reflect the brain's ability to pay attention to external information as well as its ability to process memories. Cheng's research (19) shows that healthy full-term newborns produce different MMR amplitudes in response to different emotional prosodies (happy/fearful), and higher MMR amplitudes with negative (fearful) prosodies. Our previous study (20) showed that the MMR amplitude of neonates with brain injury decreased and was related to poor neurodevelopmental outcomes at 30 months (20). However, the previous study didn't analyze the relationship between MMR amplitude and the risk of ASD. Another ERP study has indicated that 5–11 years old children with ASD showed EEG evidence of delayed speed of semantic processing and limited integration with mental representations (21). While, did these children at risk already have abnormalities in processing emotional prosody at birth? There is lacking of knowledge about this.

Therefore, we applied auditory event related potential (AERP) in IDMs, who were at risk of ASD, to explore the change of MMR under the stimulation of different emotional prosodies and to analyze whether the change of MMR in neonates was an early index predicting the development of ASD in childhood.

## Materials and Methods

### Participants

Full-term newborns admitted to the pediatric neonatal ward of Peking University First Hospital from December 1, 2017 to April 30, 2019 were selected for the study. They were divided into the IDM group and the control group. Inclusion criteria for the IDM group: (1) Their mothers met the diagnosis of gestational diabetes, with the diagnostic criteria being: Pregnant women should be offered OGTT between 24–28 weeks using a standardized 2-h 75-g oral glucose tolerance test with fasting plasma glucose (FPG), 1-h plasma glucose (PG), 2-h PG (III-B). Gestational diabetes mellitus is diagnosed if one value is met or exceeded: i. FPG ≥5.1 mmol/L; ii. 1-h PG ≥10.0 mmol/L; iii. 2-h PG ≥8.5 mmol/L (22); (2) Gestational age ≥37 weeks and birth weight above the 10th percentile for the same gestational age and sex; (3) Passed otoacoustic emission (OAE) and automated auditory brainstem response (AABR) examinations; (4) Vital signs for all children were stable at the time of examination; (5) No sedation was applied within 48 h prior to the event-related potential data collection. Inclusion criteria for the control group (non-IDM group): (1) Full-term newborns admitted to the ward in the same period without severe diseases (sepsis, pneumonia, hyperbilirubinemia, congenital heart disease, necrotizing enterocolitis); (2) Gestational age ≥37 weeks and birth weight above the 10th percentile for the same gestational age and sex; (3) Passed otoacoustic emission (OAE) and automated auditory brainstem response (AABR) examinations; (4) Vital signs for all children were stable at the time of examination; (5) No sedation was applied within 48 h prior to the event-related potential data collection.

#### Exclusion Criteria

(1) The mother has conditions such as autoimmune diseases, hypothyroidism/hyperthyroidism, hypertension during pregnancy, or taking medicines except folic acid, multivitamin and calcium tablet; (2) History of perinatal hypoxic asphyxia; (3) Brain injury such as intraventricular hemorrhage of Grades III-IV, stroke, meningitis, neonatal seizure; (4) Congenital genetic metabolic disease; (5) Unstable vital signs combined with severe diseases such as respiratory failure and shock, requiring the application of treatment such as respiratory support, vasoactive drugs.

Informed consent was signed by the parent or legal guardian of the neonates to approve the use of clinical information and EEG data for scientific purpose. The research protocol was approved by the Ethics Committee of Peking University First Hospital.

### Stimuli and Procedure

With reference to Cheng et al. study (19), the syllables “da-da” spoken by a young woman representing negativity (fear, anger), positivity (happiness) and neutrality were used as stimuli. The duration of each syllable was 350 ms.

The experiment was conducted in a soundproof room when the neonate was in relatively stable condition during hospitalization and during the natural sleep state half an hour after feeding. Emotional prosodies were played through a loudspeaker (HiFier-010A, Shenzhen, China), and EEG information was collected during the experiment. The sound intensity was 57–62 dB with an average of 59 dB, and the noise intensity of the surrounding environment was ≤ 30 dB.

The emotional prosodies were presented using an oddball paradigm, in which deviant stimuli (fearful, angry or happy prosody) were occasionally presented (20%) mixed among a series of repetitive standard stimuli (neutral prosody, 80%). The interval between stimuli ranged from 450 to 850 ms.

**Figure 1:**
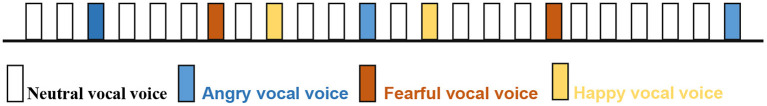
The pattern of vocal voice broadcasting.

The experiment lasted for about 60 min on average. In this study, there were 6 blocks, each with 600 stimuli, including 480 “standard stimuli” and 120 “deviant stimuli”. The two types of stimuli were played randomly (Figure 1).

### ERP Recording

An EEG recorder (Neusen.U, Borecon) was used to extract event-related potentials (ERP) according to the international standard 10–20 system electrode placement method. This was completed by acquiring EEG signals from F3 and F4 (frontal area), C3 and C4 (central area), P3 and P4 (parietal area) after different emotional voice stimuli while playing the voice signal. Electrodes were all Ag-AgCl disc electrodes with impedance <5 kQ. The EEG signals obtained after different emotional voice stimulation were extracted from the ERP data using EEGLAB with Matlab software. EEGs were filtered (1 to 30 Hz), segmented (−200 to 800 ms), baseline corrected (−200 to 0 ms), and trials with amplitudes exceeding ± 150 μV were excluded. MMR wave amplitude and latency were used to determine the subject's ability to discriminate between different emotional prososdies (angry, fearful, and happy vocal voice). Higher amplitude indicated that the brain was better at discriminating between such stimuli. Shorter latency indicated that the brain processed emotional information faster.

### Neurodevelopment Assessment

Neurodevelopmental evaluations were performed using the Ages and Stages Questionnaire (ASQ) for all the enrolled children at 3, 6, 9, 12, 18 and 24 months postpartum and the Modified Checklist for Autism in Toddlers (M-CHAT) was tested at 24 months. ASQ-3, which covers five domains (communication, gross motor, fine motor, problem-solving, and personal-social), can be completed at home (23). Each domain represents a developmental milestone for that specific age group. Infant who scored higher than the cutoff was normal; individual who scored near the cutoff was borderline and required further evaluation; individual who scored below the cutoff was considered to have developmental abnormalities (24). ASQ-SE focuses on the emotional development and mental health of children. A total score equal to or above the cutoff indicated that the infant was at risk for social-emotional disorders and was recorded as a failed test. M-CHAT is a commonly used screening scale for autism in infants and toddlers in China at present (25). There are 23 items on the scale. The screening was considered failed if items 11, 18, 20, and 22 were scored “3” and other items were scored “0”. The infant was considered to be at risk for autism or other developmental disorders if two or more among items 2, 7, 9, 13, 14, and 15 were scored as “3”, or if there were three or more items out of all items on the list that were scored as “3”. A higher total score reflects a greater risk for ASD (26). A total score of ≥17 was considered failing to pass the test (see the attachment). Finally, 43 newborns (23 in IDM group and 20 in control group) were followed up to 24 months and ASQ-3, ASQ-SE and M-CHAT evaluations were performed by then.

### Statistical Analysis

Statistical analyses were performed using SPSS 21.0 software. Repeated measures ANOVA was performed on the amplitude of ERP, with group (neonates born to a diabetic mother vs. not born to a diabetic mother) as the between-subject factor, emotional prosodies (negative: fearful and angry, positive: happy) and electrode position (F3, F4, C3, C4, P3, and P4) as within-subject factors. Significance level was set at *p* < 0.05. Effect sizes of independent variables were assessed using biased η2. Bonferroni correction was used for multiple comparisons. Greenhouse-Geisser correction for degrees of freedom was used.

## Results

### General Demographics

A total of 50 neonates were enrolled in this study. 7 cases were excluded due to poor data caused by crying of the infants, and 43 cases were included ultimately. Among them, there were 23 cases in IDM group (15 males and 8 females) and 20 neonates in the control group (10 males and 10 females). The IDM and the control groups did not differ by sex, gestational age, birth weight, time of ERP acquisition and neurodevelopmental assessment, and maternal age and education. Median maternal age was 33 years (ranged from 26–45) in the IDM group and 32 years (ranged from 24–44) in the control group. In our study, 87% of the parents (20/23) in IDM group and 90% of the parents (18/20) in control group achieved a university degree or above. Besides, cranial ultrasound was done in all infants of this study. 38 of them are normal and 5 of them got a subependymal hemorrhage at absorption period which didn't affect the neurodevelopment outcomes. The general demographics in both groups is summarized in Table 1.

**Table 1:** Participant demographics.

***N* (%) or mean ±SD**	**Control** ***N*** **= 20**	**IDM** ***N*** **= 23**	***P*** **-value**
**Child characteristics**			
Male/Female	10/10	15/8	0.365
Gestational age (w)	39.5 ± 0.9 (37–40)	38.7 ± 0.9 (38–41)	0.954
Birth weight (g)	3210 ± 520 (2550–4150)	3313 ± 634 (2600–3980)	0.618
ERP acquisition (d)	5.8 ± 3.1 (2–10)	4.3 ± 2.1 (2–14)	0.274
Neurodevelopmental assessment (m)	24.0 ± 0.8 (23–25)	24.2 ± 0.7 (23–25)	0.299
**Maternal characteristics**			
Age (y)	31.9 ± 5.6 (24–44)	30.8 ± 5.2 (26–45)	0.622
Education (college and above)	18 (90)	20 (87)	0.756

*N (%), number (percent of total); SD, standard deviation; w, week; g, gram; d, day; m, month; y, year*.

#### ERPs

##### MMR Amplitude

**Figure 2:**
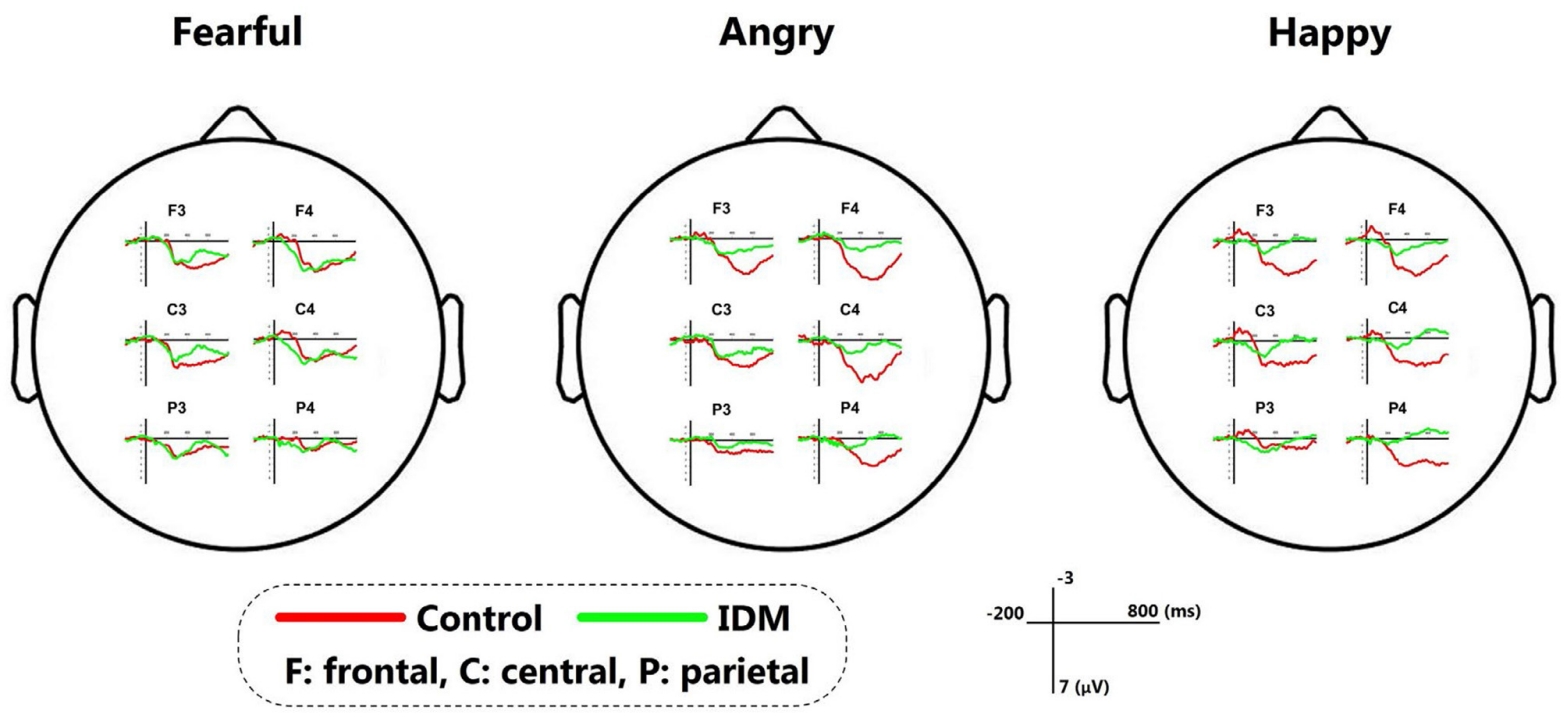
The MMR of IDM and control group stimulated by fearful/angry/happy prosodies.

The main effect of group was not significant [F(1, 42) = 3.069, *p* = 0.108, η^2^ = 0.218]: though the MMR amplitudes were smaller in the IDM group (1.05 ± 0.22 μV) compared to the control group (1.63 ± 0.23 μV). The main effect of emotion valence was significant [F(3, 126) = 6.487, *p* = 0.013, η^2^ = 0.684]: fearful prosody (1.75 ± 0.21 μV) evoked larger MMR amplitudes than happy and angry prosodies (1.38 ± 0.18 μV, 1.31 ± 0.29 μV). The main effect of electrode position was significant [F(5, 210) = 6.911, *p* = 0.012, η^2^ = 0.832]. In general, the MMR amplitudes declined from front to back (F3 = 1.33 ± 0.29 μV, F4 = 2.24 ± 0.23 μV, C3 = 1.59 ± 0.21 μV, C4 = 1.89 ± 0.34 μV, P3 = 0.32 ± 0.28 μV, P4 = 0.66 ± 0.36 μV). The most important finding was the significant interaction between emotion prosody × electrode position × group [F(1, 42) = 20.020, *p* = 0.001, η^2^ = 0.645]: while fearful prosody evoked larger MMR amplitudes at F3 in the control group (3.47 ± 0.47 μV) than the IDM group (1.23 ± 0.64 μV). There was no significantly statistic difference between other emotions in two groups. Detailed results are shown in Table 2 and Figure 2.

**Table 2:** Comparison of MMR amplitudes produced by two groups at different electrode positions and with different emotional prosodies (± S, μV).

	**Electrode position**	**MMR Amplitude**		
		**Angry**	**Fearful**	**Happy**
	F3	−0.32 ± 0.78	1.23 ± 0.64	0.29 ± 0.62
	F4	1.73 ± 0.64	2.43 ± 0.55	1.50 ± 0.80
IDM	C3	0.61 ± 0.61	1.48 ± 0.82	0.95 ± 0.53
	C4	1.37 ± 0.64	2.03 ± 0.50	1.54 ± 0.73
	P3	1.75 ± 0.67	0.65 ± 0.71	0.39 ± 0.87
	P4	1.76 ± 0.33	1.41 ± 0.71	0.93 ± 0.60
	F3	2.67 ± 1.01	3.47 ± 0.47	2.58 ± 0.89
Control	F4	3.51 ± 0.77	3.35 ± 0.77	3.65 ± 0.48
	C3	1.56 ± 0.73	2.9 ± 0.40	2.31 ± 0.60
	C4	1.79 ± 0.76	2.57 ± 0.86	2.88 ± 0.70
	P3	−0.92 ± 0.71	−0.11 ± 0.77	−0.60 ± 0.71
	P4	0.25 ± 0.71	−0.44 ± 0.72	0.17 ± 0.86

#### MMR Latency

The main effect of group was significant [F(1, 42) = 14.607, *p* = 0.003, η^2^ = 0.594]: the MMR latency were shorter in the IDM group (300 ± 9 ms) compared to the control group (346 ± 13 ms). Interaction between emotion prosody × electrode position × group showed: (1) fearful prosody evoked longer MMR latency at F3 in the control group (422.5 ± 31.9 ms) than the IDM group (288.2 ± 44.5 μV), [F(1, 42) = 65.316, *p* = 0.000, η^2^ = 0.867]. (2) angry prosody evoked longer MMR latency at F3/F4 in the control group (F3: 419.8 ± 38.2 ms, F4: 453.1 ± 25.6 ms) than the IDM group (284.0 ± 28.3 μV, F4: 329.0 ± 33.1 ms), [F3: F(1, 42) = 10.874, *p* = 0.008, η^2^ = 0.521; F4: F(1, 42) = 8.311, *p* = 0.016, η^2^ = 0.454]. There was no significantly statistic difference between two groups under happy prosody.

#### Neurological Prognosis

The results showed that no infant failed ASQ-3 and ASQ-SE at 24 months and no significant statistic difference was found between two groups in five domains (IDM vs Control: Communication: 52.8 ± 5.7 vs 51.5 ± 7.7, *p* = 0.074; Gross motor: 55.0 ± 4.3 vs 57.7 ± 3.9, *p* = 0.887; Fine motor: 57.8 ± 3.6 vs 55.4 ± 5.6, *p* = 0.308; Problem solving: 52.2 ± 5.1 vs 51.9 ± 6.0, *p* = 0.399; Personal-social: 54.4 ± 3.9 vs 53.1 ± 6.0, *p* = 0.058) and ASQ-SE scores (IDM vs Control: 27.3 ± 9.0 vs 23.5 ± 14.3, *p* = 0.114). There was no abnormality in the M-CHAT scores of two groups. No significant statistic difference was found between gestational age/sex and M-CHAT scores (*p* = 0.176, *p* = 0.663).

**Figure 3:**
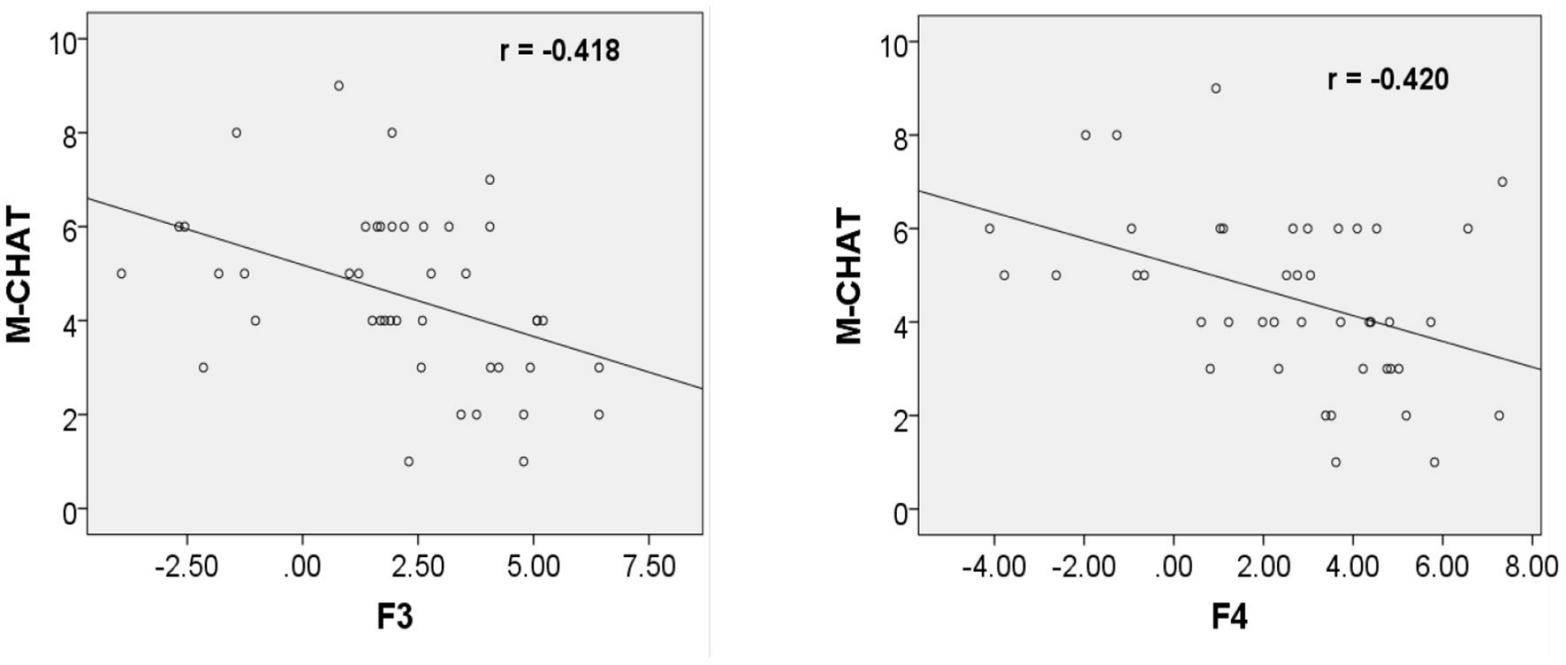
Correlation between ERP amplitudes at F3/F4 in response to fearful prosody and M-CHAT score at 24 months.

However, the ERP amplitudes at the frontal electrodes in response to fearful prosodies were negatively correlated with M-CHAT score (F3: r = −0.418, *p* = 0.005; F4: r = −0.420, *p* = 0.005; Figure 3).

## Discussion

Abnormal maternal glucose metabolism during pregnancy leads to alterations in the intrauterine environment that can interfere with fetal brain development. Existing researches have found that the shape and volume of the hippocampus in IDM may be affected by intrauterine hyperglycemia (27). The hippocampus is a subcortical structure critical for learning, memory and emotion regulation (28), and hippocampus impairment may affect neurological function either directly or by breaking off neural connections. Increased risk of attention deficits, poor gross and fine motor skills, and reduced expressive language skills may occur during growth (29), but there are usually no specific neurological symptoms in the neonatal period or even in infancy. Neither early classical structural brain examinations such as MRI nor functional examinations like conventional EEG can detect abnormalities like that especially in neonates (30–37). In this study, we found that emotional prosody discrimination was affected in IDM in the early postnatal period by ERP, as evidenced by a significant decrease in ERP amplitude in response to fearful prosody at F3 and shorter latency in response to fearful and angry prosodies. Follow-up revealed a negative correlation between fearful prosody wave amplitude at F3/F4 in the early postnatal period and M-CHAT scores at 2 years of age despite all of them act normal by then.

Limited studies (38, 39) showed changes of ERP in IDMs. In Cordón's study (39), impairments of emotional face recognition were observed in ERPs from 3 to 4 years old children born to diabetic mothers (CDM). It showed that CDM displayed longer latencies and lower amplitude when processing the fearful and angry expressions, while not at happy procession. In our study, decreased amplitudes of MMR were also found in fearful and angry prosodies, though it was statistically significant in fearful prosody at F3. The different processing between negative and positive information may due to less negative stimuli exposure than happy stimuli during early development, and infants are more familiar to the happy prosody. Another finding in this study was that IDM produced significantly shorter ERP latency in response to different emotional prosodies compared to the control, which was inconsistent with the above research. While in deRegnier et al. study (40), it also showed shorter latency of P2 in ERP of IDM when recognizing mother's voice from stranger's. In general, the latency reflects the speed at which the brain processes information and often correlates with the maturation of the myelination of the nerves (41). Though shorter latency of ERP was found in both studies, it doesn't mean more mature of the brain in IDMs. Because longer duration of information processing was observed in control both in our and deRegnier's studies, which means greater continuous processing capacity. Besides, there's no relationship between short latency and better neurodevelopmental outcome at 24 months old in our study. This finding needs to be replication, admitting more subjects, and sleep cycle should also be monitored and discussed synchronously, which may affect the result of ERP, either.

The current study (42) shows the role of ERP in prediction of neurological outcome in infants and children. deRegnier et al. study (40) has shown that IDM produce a different pattern of ERP compares to controls and the altered pattern may be associated with cognition outcome at 1 year of age (as evidenced by a decrease in mental development index). A prospective study in Singapore (43), which performed AERP in IDM at 6 months and 18 months, showed that left hemisphere MMR wave amplitude in the brain at 6 months of age was significantly associated with BSID-III cognitive scores, i.e., the higher the MMR wave amplitude, the higher the cognitive score. Our study used ASQ as a screening method and did not find any children with developmental abnormalities. It may be related to the fact that glycemic control was satisfied in the mothers of the IDM in this study during pregnancy and the newborns were born without complications such as hypoglycemia. The IDMs in this study showed a slightly decrease in ERP amplitude, it may indicate that the small effect of maternal hyperglycemia on the brain development. Therefore, further classification of IDM according to maternal glucose during pregnancy and serum iron levels would be beneficial to explore the effect of maternal glucose levels during pregnancy on IDM neurodevelopment.

Autism is a severe but not rare developmental mental disorder that may begin as early as infancy (44). The onset of autism is characterized by early onset, complex symptoms and a poor prognosis. Although the etiology of autism is not yet clear and there is no satisfactory treatment, early diagnosis and treatment have an important role on the prognosis of children with autism at pre-school age. The main screening scale commonly used in China at this stage is the M-CHAT. It has a high degree of authenticity, specificity and reliability (25). The screening in our study did not find any children with ASD, but analysis showed a negative correlation between ERP amplitude elicited by fearful prosody at F3/F4 and their scores in the early postnatal period. It is unclear if this means that the risk of developing ASD is higher when ERP amplitude is lower, but it is an extremely interesting phenomenon. Further follow-up and large sample size are needed to explore this.

### Limitations

(1) There was no child with developmental retardation at 24 months old. This could be attribute to the small number of subjects included in this study and most mothers in this study had satisfactory glucose control. However, we still observed difference in ERP between the IDM and control group. Subsequent studies may admit more subjects with poor maternal glucose control, and the predictive role of ERP in IDM neurodevelopmental outcome will be discussed further. (2) Due to the COVID-19 epidemic, this study used ASQ and M-CHAT screening tests, which are easy for parents to complete at home, instead of diagnostic scales for infant neurodevelopment. This may be more convenience for parents, but not as objective as scales done by doctors. In addition, some emotional and behavioral problems, such as attention deficit hyperactivity disorder couldn't be diagnosed such early, and long term follow-up is needed for these children.

## Conclusion

This study found that Infants of diabetic mothers had different procession in emotional prosodies, especially in fearful prosody during neonatal period, as evidenced by a shortened latency and decreased ERP amplitude. Though all children in this study passed neurodevelopment assessment at 24 months old. Infants with low ERP amplitude under fearful prosody at F3/F4 had high M-CHAT scores at 24 months, which should be alerted to the risk of ASD.

## Data Availability Statement

The raw data supporting the conclusions of this article will be made available by the authors, without undue reservation.

## Ethics Statement

The studies involving human participants were reviewed and approved by Ethics Committee for Clinical Research of Peking University First Hospital. Written informed consent to participate in this study was provided by the participants' legal guardian/next of kin.

## Author Contributions

All authors listed have made a substantial, direct, and intellectual contribution to the work and approved it for publication.

## Funding

This study was funded by Beijing Municipal Science & Technology (Z191100006619049 to XH), National Key Research and Development Program of China (2021YFC2700700 to XH), Capital's Funds for Health Improvement and Research (2022-3-40715 to XH), Beijing Municipal Science & Technology Commission (Z211100002921050 to LL), Young Scientists Fund of the NSFC (82101806 to LL), and Scientific Research Seed Fund of Peking University First Hospital (No. 2019SF12 to RZ).

## Conflict of Interest

The authors declare that the research was conducted in the absence of any commercial or financial relationships that could be construed as a potential conflict of interest.

## Publisher's Note

All claims expressed in this article are solely those of the authors and do not necessarily represent those of their affiliated organizations, or those of the publisher, the editors and the reviewers. Any product that may be evaluated in this article, or claim that may be made by its manufacturer, is not guaranteed or endorsed by the publisher.
