# Supplementary material for: Emotional Prosodies Processing and Its Relationship With Neurodevelopment Outcome at 24 Months in Infants of Diabetic Mothers
Source: Front Pediatr. 2022 May 18;10:861432. doi: 10.3389/fped.2022.861432 (PMC9159506; doi:10.3389/fped.2022.861432)
Supplement: Supplementary file 1 [file Data_Sheet_1.pdf]

M-CHAT (Modified Infant Autism Scale Chinese Revised Version)

Name_____ Sex____ Date of birth____ Age____

Please selected the option that best reflects your child's situation (select only one option). Try not to leave anything out.

1.Does your child like to be rocked and bounced on your knees?	Never 3	Occasionally 2	Sometimes 1	Often 0
2.Is your child interested in other children?	Never 3	Occasionally 2	Sometimes 1	Often 0
3.Does your child like climbing up and down stairs?	Never 3	Occasionally 2	Sometimes 1	Often 0
4.Does your child like playing peekaboo game?	Never 3	Occasionally 2	Sometimes 1	Often 0
5.Does your child pretend to do things? eg. Talking on the phone or taking care of a doll or pretending to do something else?	Never 3	Occasionally 2	Sometimes 1	Often 0
6.Has your child ever pointed his/her index finger at something and asked for it?	Never 3	Occasionally 2	Sometimes 1	Often 0
7.Has your child ever pointed his/her index finger at something and showed interest in it?	Never 3	Occasionally 2	Sometimes 1	Often 0
8.Does your child play with small toys (such as cars or blocks) properly, rather than putting them in his/her mouth, fiddling with them or throwing them away?	Never 3	Occasionally 2	Sometimes 1	Often 0
9.Has your child ever show you (parents) anything?	Never 3	Occasionally 2	Sometimes 1	Often 0
10.Does your child look into your eyes for more than one second or two?	Never 3	Occasionally 2	Sometimes 1	Often 0
11.Does your child ever seem to be sensitive to noise (eg. covering his/her ears) ?	**Never 0**	**Occasionally 1**	**Sometimes 2**	**Often 3**
12.Does your child smile back when he/she look at your face or smile?	Never 3	Occasionally 2	Sometimes 1	Often 0
13.Will your child imitate you? (eg. If you make a face, will your child imitate it?)	Never 3	Occasionally 2	Sometimes 1	Often 0
14.When you call your child's name, does he/she respond?	Never 3	Occasionally 2	Sometimes 1	Often 0
15.If you point to a toy across the room, will your child look at it?	Never 3	Occasionally 2	Sometimes 1	Often 0
16.Can your child walk?	Yes 0		No 1	
17.Will your child watch what you are looking at?	Never 3	Occasionally 2	Sometimes 1	Often 0
18.Does your child make special finger movements near his/her face?	**Never 0**	**Occasionally 1**	**Sometimes 2**	**Often 3**
19.Does your child try to attract you to his/her own activities?	Never 3	Occasionally 2	Sometimes 1	Often 0
20.Have you ever suspected that your child has a hearing problem?	**Never 0**	**Occasionally 1**	**Sometimes 2**	**Often 3**
21.Does your child understand what other people say?	Never 3	Occasionally 2	Sometimes 1	Often 0
22.Does your child sometimes stare aimlessly or walk aimlessly?	**Never 0**	**Occasionally 1**	**Sometimes 2**	**Often 3**
23.Does your child look at your face when they encounter unfamiliar things and watch your reaction?	Never 3	Occasionally 2	Sometimes 1	Often 0

Evaluator________ Evaluation date _____________

If the answer of items 11,18,20,22 of the scale is '3', and the answer of the remaining items is '0', the child is consider as failing to pass the test. If '3' for 2 or more items of '2,7,9,13,14 and 15' or '3' for 3 or more of all items, the risk of autism or other development disorders is considered. A total score of ≥17 is considered as failing to pass the test.
